# Round Window Niche and Membrane Dimensions: A Systematic Review

**DOI:** 10.3390/audiolres15040090

**Published:** 2025-07-23

**Authors:** Mathieu Marx, Pauline Nieto, Olivier Sagot, Guillaume de Bonnecaze, Yohan Gallois

**Affiliations:** 1 Service d’Otologie, Otoneurologie et ORL Pédiatrique, Hôpital Pierre-Paul Riquet, CHU Toulouse Purpan, 31000 Toulouse, France; 2 Brain & Cognition Research Centre, UMR 5549, Université Toulouse III, 31000 Toulouse, France; 3 Institut d’Anatomie, Université Toulouse III, 31000 Toulouse, France

**Keywords:** round window, inner ear, drug delivery, vibroplasty

## Abstract

**Background/Objectives**: To review the dimensions of the round window region (round window niche, bony structures surrounding the niche, and the membrane itself). **Methods**: Medline, EMBASE, Cochrane Library, and Google Scholar databases were searched by two independent reviewers. Anatomical and radiological studies on the round window region were screened. Studies reporting at least one dimension for the round window (RW) niche and/or the RW membrane were included. **Results**: Sixteen studies met the inclusion criteria (13 anatomical and 3 radiological studies) for a total number of 808 temporal bones with at least one dimension reported. The structures measured varied across the different studies with 12 reporting RW membrane dimensions (area and/or at least one distance), 8 detailing RW niche dimensions (height, width or depth) and 6 which measured at least one element of the RW bony overhangs (posterior or anterior pillar, RW tegmen). Surface area of the RW membrane varied between 0.32 mm^2^ and 2.89 mm^2^, with a minimum dimension (minimum diameter or height or width) comprising between 0.51 mm and 2.1 mm. When the bony overhangs surrounding the membrane were not considered, the minimum diameter was between 1.65 mm and 1.97 mm. **Conclusions**: The dimensions of the RW region are intrinsically variable, but the heterogeneity of the measurements reported also contributes to these variations. Posterior pillar, RW tegmen, anterior pillar, and their relative development probably account for a large part of this variability. The future RW membrane devices should be ≤1 mm in their maximum dimension, whether or not individually tailored, to fit most of the RW membranes.

## 1. Introduction

Over the last two decades, advances in the treatment of hearing loss and other inner-ear disorders have renewed interest in the round window (RW) and its anatomy. Indeed, the spread of cochlear implantation with the emergence of atraumatic cochlear implant surgery in the 2000s generated multiple valuable studies on the best site for CI entry into the cochlea [1,2]. Likewise, the first description by Colletti et al. [3] of the direct placement on the RW membrane of an active middle ear implant (i.e., the floating mass transducer (FMT) of the Vibrant Soundbridge) was followed by numerous publications on the results of RW vibroplasty [4] (see Beltrame for review) [4]. This alternative mechanism for stimulating the cochlea has been shown to provide good hearing outcomes on average in the case of mixed or conductive hearing loss [3,4]. However, these results were dependent on the quality of the coupling to the RW membrane and the stability of the device over time, two issues that significantly reduced the interest of this strategy for other authors. Furthermore, recent developments in inner-ear drug delivery led researchers to study access to the RW membrane and evaluate its permeability. Similarly, several reports raised concerns due to the recurrent presence of an additional mucosal fold covering the membrane itself (up to 55% according to Stewart & Belal, 1981 [5]) and protruding bony overhangs, which might impede the diffusion of treatments across the RW.

From the seminal descriptions [5,6,7], it was established that the anatomy of the RW area is highly variable across individuals, even though some reliable landmarks could be consistently identified. The recent advent of endoscopic ear surgery renewed and completed these reports, as it allows an improved visualization of the structures contained in the middle ear cleft in general. More specifically, a simple 0° endoscope substantially improves the visualization of the elements located in the retro-tympanum, including the RW niche. The RW niche stands in a posteroinferior position with respect to the external auditory canal and has been described as a triangular or quadrangular cavity with the RW membrane at its apex. The RW membrane is partially covered by bony elements of the niche, commonly gathered under the label of “bony overhang”, but actually composed of the posterior pillar posteriorly, the tegmen superiorly, as well as the anterior pillar anteriorly. An additional bony crest, the crista fenestra, lies on the floor of the RW membrane. The inferior wall of the RW niche corresponds to the fustis, a dense bony plate surrounded by tiny air cells, and the area concamerata. Posterior and anterior pillars extend in an inferior direction, respectively, through the subiculum and the finiculus (also named sustentaculum in Proctor’s pioneering paper). In 75% to 80% of temporal bones, a tunnel between the fustis and the finiculus runs inferiorly to the RW towards the petrous apex [8,9] and is called the subcochlear canaliculus. The variability in the size and shape of pillars and tegmen, as well as the RW membrane itself, has repeatedly been reported in most anatomical studies on the RW area.

The aim of the present literature review was to focus on the dimensions of the RW niche, the membrane, and the surrounding structures of the RW niche (pillars, fustis). On the basis of these dimensions, future RW devices, whether used for drug delivery or mechanical stimulation, could be developed.

## 2. Materials and Methods

This systematic review was performed using the “Preferred Reporting Items for Systematic Review and Meta-Analysis” PRISMA statement with the checklist adapted to anatomical reviews by Yammine (2014) [10] when introducing the concept of evidence-based anatomy. This checklist allows a systematic assessment of anatomical descriptive cross-sectional studies [10]. Publications in English were searched in several indexed resources (Medline via PubMed, EMBASE, Cochrane, Google Scholar) between 5 and 31 July 2024 using various combinations of the following keywords: round window—round window niche—dimensions—size. No date restrictions were used. In the PubMed database, we used the medical subject heading (MeSH) “round window OR round window niche” with the search builder and added the additional text qualifier “AND size OR dimension”. Two filters were applied: “Humans” and “English”. The same word combinations were used in the other databases. The “references” section of every relevant article and all full-text articles eligible for inclusion were also carefully examined and evaluated. This study’s protocol was not registered in any online database. Inclusion criteria were studies written in English reporting at least one dimension measured for the RW membrane and/or the bony structures of the RW niche (pillars, tegmen, fustis). These dimensions could include length, width, height, and thickness measurements and were described as means associated with standard deviation or distribution range. These studies could be performed on living patients or cadaveric specimens using anatomical or radiological measures. Although some histological studies were highly informative [6,11,12], they were excluded because they can only provide two-dimensional measurements on a limited number of sections, without a full visualization of the RW area, as pointed out by Okuno & Sando [13]. Conference abstracts, animal studies, and studies that reported irrelevant data were excluded. Two authors of the present systematic review (MM and PN) independently performed the comprehensive literature search, screened the articles according to the inclusion criteria, and extracted the studies to include. Disagreements between the two reviewers about a particular study to include were resolved by consensus. During the full-text evaluation and data collection process, all data were extracted using a standard data worksheet which included all of the following categories: Year of publication—Authors—Publication title—Measurement technique (anatomical on cadaveric temporal bones or radiological or both)—Structure(s) and Dimension(s) measured. The Anatomical Quality Assurance (AQUA) tool [11] was used independently by two authors of the present review (MM and PN) to assess the risk of bias for each article. This systematic review of published articles on RW anatomy did not require any ethics approval or informed consent.

## 3. Results

**Figure 1:**
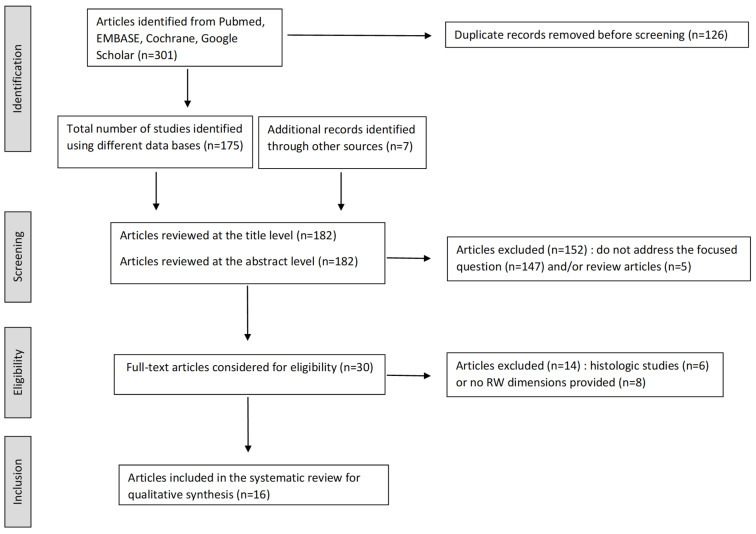
Flow diagram of the articles included in the systematic review.

A total of 182 articles were retrieved (175 using the different databases and 7 additional hand-searched articles through reference lists) after removal of duplicate records, while 30 remained for full-text review after eliminating subject and review articles. Fourteen articles were further excluded because they did not provide any measurements of the RW area dimensions (*n* = 8) or reported histological measurements (*n* = 6). Thus, 16 articles were reviewed (see Flow Diagram Figure 1) for a total number of 808 temporal bones with at least one distance measured at the RW level. Thirteen studies reported anatomical measurements made on temporal bone specimens. Among these, 1 study made radio-anatomical correlations in the same specimen using Computed Tomography (CT) scan [12]. Three studies reported CT scan measurements of the RW region in patients (*n* = 2; Cohen et al. [14]; Matin et al. [15]) or cadaveric specimens (*n* = 1; Cornwall et al. [16]) using micro-CT scan.

Different types of dimensions of the RW membrane were reported (see Table 1): surface area in mm^2^ [15,17,18,19,20,21,22], maximum diameter [12,17], maximum and minimum diameter [19,22], width [14], height and width [20,21,23]. Surface area varied between 0.04 mm^2^, measured before any drilling of the bony overhangs [18], to 2.89 mm^2^, considering the whole RW membrane, without bony overhangs [22]. Minimum diameter was measured between 0.9 mm and 2.1 mm [19], whereas maximum diameter varied between 1.32 mm and 2.35 mm [19]. Height ranged from 0.51 mm [23] and 2.3 mm [21] while width was between 0.51 mm and 2.04 mm [23].

At the level of the RW niche, the maximum transverse diameter (or width) varied between 0.6 mm [5] and more than 3 mm [24]. When height was measured, the maximum values were reported between 1.6 mm [5] and 2.4 mm [21]. Matin et al. measured the depth between the entry of the niche to the RW membrane (mean: 1.35 mm) [15]. The thickness of the RW tegmen was measured in three anatomical studies, and its mean varied between 0.66 (SD: 0.16) [24] and 2.1 mm (minimum: 1.9; maximum: 2.4) [25]. Measurements made on the posterior pillar are presented in Table 1. Only a few studies have examined the length of the anterior pillar: Stewart & Belal [5] found a mean length of 1.4 mm (minimum: 0.9; maximum: 2.3), while Shakeel et al. [25] reported a mean length of 4.0 mm (minimum: 3.3; maximum: 4.95). It is unclear whether the finiculus was also considered in Shakeel’s study [25]. There was no study including measurements of the fustis, although this structure is reported as constant. The risk of bias, estimated based on the AQUA tool [11], was considered to be high in all studies using CT scan measurements, intrinsically limited by their spatial resolution on millimetric anatomical structures. The studies made on anatomical specimens using a mold technique or a millimeter-calibrated scale were classified as low risk of bias. This risk was defined as unclear, where the reliability of the method used to conduct the measurements was unclear [22].

**Table 1:** Studies included in the systematic review and the dimensions reported. ^†^ These two studies used the same temporal bone specimen. The measurements of the RW niche differ between the two because the first one reported the dimensions at the entry of the RW niche, and the second one reported the dimensions at the apex, in close contact with the RW membrane. * Measurements of the RW membrane were made from the scala tympani side, considering its floor in Liang et al. [22] and the tip of crista fenestra in Atturo et al. [19]. ** Karkas et al. [12] was a radio-anatomical study, performed on the same temporal bone specimens. ^§^ Mehanna et al. [21] provide two data sets: one obtained on cadaveric temporal bones, and one on pre-operative CT scans performed in future cochlear implant recipients.

	RW Membrane	RW Niche	Posterior Pillar	RW Tegmen
Stewart & Belal [5]; *n* = 68 (cadaveric temporal bones, CTB) (risk of bias ROB: high)		Transverse diameter: 1.5 (min: 0.6; max: 2.8) Vertical diameter: 1.2 (min: 1.2; max: 1.6	Length: 2.8 (min: 2.0; max: 4.0)	Length: 1.1 (min: 0.5; max: 1.8)
Okuno & Sando [13]; *n* = 40 (CTB) (ROB: high)	Area: 2.29 mm^2^ (SD: 0.42)			
Takahashi et al. [24] ^†^; *n* = 5 (CTB) (ROB: high)		Maximal diameter: 2.98 (SD: 0.23) Depth: 0.66 (SD: 0.16)		Thickness: 0.66 (SD: 0.16)
Takahashi et al. [17] ^†^; *n* = 5 (CTB) (ROB: high)	Area: 2.98 mm^2^ (min: 2.51; max: 3.72)	Maximal diameter: 2.32 mm (min: 2.13; max: 2.53) Length: 2.08 mm (min: 1.86; max: 2.43) Width: 1.76 mm (min: 1.61; max: 1.89)		
Roland et al. [18]; *n* = 15/30 (CTB) (ROB: low)	Before drilling overhang, area: 0.32 mm^2^ (min: 0.04; max: 0.61) After drilling overhang, area: 0.72 mm^2^ (min: 0.46; max: 1.16)			
Singla et al. [26]; *n* = 50 (CTB) (ROB: low)		Maximum height: 1.62 (SD: 0.77) Maximum width: 1.15 (SD: 0.39)		
Atturo et al. [19] *; *n* = 30 (CTB) (ROB: low)	Long diameter: 1,9 mm (min: 1.32; max: 2.35) Small diameter 1.54 mm (min: 0.9; max: 2.1) Area: 2.08 (min: 0.99; max: 3.2)			
Shakeel et al. [25]; *n* = 14 (CTB) (ROB: unclear)		Vertical height: 2.02 mm (min: 1.85; max: 2.15)		Thickness: 2.1 (min: 1.9; max: 2.4)
Angeli et al. [20]; *n* = 10 (CTB) (ROB: high)	Height: 0.77 mm (min: 0.6; max: 1.04) Width: 1.41 mm (min: 1,19; max: 1.71) Area: 0.91 mm^2^ (min: 0.54; max: 1.29) Area after drilling crista fenestra: 1.42 mm^2^ (min: 0.83; max: 2.02)			
Karkas et al. [12] **; *n* =10 (CTB) (ROB: high)	Maximal diameter: 1.2 mm (SD: 0.2)			Thickness: 1.3 mm (SD: 0.2)
Jain et al. [23]; *n* = 34 (CTB) (ROB: low)	Maximum height: 0.69 (min: 0.51; max: 1.27) Maximum width: 1.16 (min: 0.51; max: 2.04)			
Mehanna et al. [21] ^§^; *n* = 20 (CTB) (ROB: high)	Maximum height: 1.53 mm (min: 0.9; max: 2.3) Maximum width: 1.18 (min: 0.8; max: 1.65) Area: 1.52 mm^2^ (min: 1.24; max: 1.52)		Length: 2.09 (min: 1.7; max: 2.37)	
Liang et al. [22] *; *n* = 14 (CTB) (ROB: unclear)	Short diameter: 1.81 mm (min: 1.65; max: 1.97) Long diameter: 2.04 mm (min: 1.87; max: 2.20) Area: 2.89 mm^2^ (2.67; max: 3.16)			
Cohen et al. [14]; *n* = 414 (CT scans) (ROB: high)	Width: 1.58 mm (min: 0.9; max: 2.7)	Width: 2.22 mm (min: 0.9; max: 3.4) Length: 1.67 mm (min: 1.0; max: 2.7)		
Matin et al. [15]; *n* = 50 (CT scans) (ROB: high)	Area: 2.93 mm^2^ (min: 1.3; max: 4.39) Volume: 4.54 mm^3^ (min:2.28; max: 6.64)	Depth: 1.35 mm (min: 0.98; max: 1.88)		Thickness: 0.56 mm (min: 0.04; max: 1.24)
Karkas et al. [12] **; *n* = 10 (CT scans) (ROB: high)	Maximal diameter: 1.5 mm (SD: 0.2)			Thickness: 1.1 mm (SD: 0.1)
Mehanna et al. [21] ^§^; *n* = 20 (CT scans) (ROB: high)		Maximal transverse diameter: 1.27 mm (min: 1.2; max: 2.3) Height: 1.52 mm (min:1.1; max: 2.4)		
Cornwall et al. [16]; *n* = 14 (CT scans) (ROB: high)	Maximum diameter: 1.3 mm (min: 1.07; max: 1.44)			

## 4. Discussion

### 4.1. Variability of the RW Area Anatomy

RW aspect variability is a recurrent finding in the literature. It involves structures composing the RW niche (pillars and tegmen) as well as the RW membrane itself. Indeed, the different dimensions reported for the RW membrane in anatomical studies may vary by a factor of 1.5 [20] to more than 2 [21,23] between the minimum and the maximum value. Similarly, the surface area of the RW membrane was highly heterogeneous between 0.32 mm^2^ before drilling the bony overhangs [18] to 2.89 mm^2^ considering the RW membrane itself [22]. The gross shape of the RW membrane is variable. Mehanna’s study [21] detailed 50% oval, 25% round, 10% triangular, 10% triangular, and one pear-shaped specimen (i.e., 5%) or Jain’s study [23] where 24 out of 34 RW membranes were described as saddle-shaped, 8 as ovoid and 2 as triangular.

This important variability could rely on several formal or content explanations. The first one lies in the methods used to perform the measurements. Among the 12 anatomical studies included, most of them used digitalized photographs treated by image software to estimate the dimensions of interest [16,17,18,19,20,21,23,24,26] while others performed direct measurements on the specimens using a mold technique [25], thin pin-gages [12], or a millimeter-calibrated scale [5]. The second reason this variability might be overestimated relates to the dimensions that were measured and where they were measured from. For instance, height and width were measured in Angeli et al. [20], whereas Atturo et al. [19] and Liang et al. [22] refer to one long and short diameter. Okuno & Sando [13] and Roland et al. [18] only reported the area covered by the RW membrane. The measurements were made from a posterior tympanotomy in studies by Roland [18] or Jain [23], while Liang et al. [22] assessed the dimensions of interest from the scala tympani after a specific preparation of the temporal bone specimens. In the latter case, the RW membrane may be fully visualized, whereas a posterosuperior part of it may be hidden by the posterior pillar and tegmen protrusion in the event a posterior facial recess approach is used.

However, the anatomical variability of this region has consistently been demonstrated and should not be discarded based only on these methodological variations. In their important study on 783 temporal bones, Toth et al. [27] described no less than 9 different types of RW niche according to the relative development of the pillars, the tegmen, and the possible obstruction of the fundus. An extremely narrow niche, due to hypertrophic bony overhangs, was, for instance, found in 11.2% of the specimens. The RW tegmen is always convex with a free edge narrower than its promontorial part and protrudes to varying degrees over the RW membrane [7,27]. The posterior pillar corresponds to a dense bony structure, thicker than its anterior counterpart, which joins the subiculum posteriorly. The anterior pillar is more variable in its presentation, ranging from a continuous and dense plate to a trabecular strip [27]. It fuses with the finiculus anteroinferiorly. In Shakeel’s study [25], the length of the anterior pillar was quite important (mean: 4.0 mm) compared to what was reported by Stewart and Belal (1.4 mm) [5] or Mehanna et al. (2.22 mm) [21]. It is plausible that the anterior pillar and the finiculus were measured altogether in the study conducted by Shakeel.

The protrusion of the pillars and tegmen was also assessed in Marchioni’s endoscopic study on the RW region [8]. In 23.7% of cases, this protrusion was limited and allowed the visualization of the RW membrane. However, in more than 75% of middle ears, the morphology or the angulation of the tegmen prevented the visualization of the RW from the external auditory canal.

### 4.2. Constant Anatomical Features: Fustis and Three-Dimensional (3D) Shape of the RW Membrane

As illustrated above, the recent multiplication of endoscopic studies has significantly improved expertise in the RW region anatomy. More specifically, they have yielded a better description of an underestimated structure, which is the fustis (meaning stick-shaped). In Proctor’s report [7], it was identified as a “solid bony column” located in the inferior and medial part of the niche. Toth et al. specified that it is the most typical bony process to occur during early fetal development of the bony RW between its creation in the 16th and 23rd week [27]. In their study, it was added that the fustis systematically leads to the crista fenestra, which is apparent from the 18th fetal week.

Marchioni et al. [9] published the first endoscopic study focused on the description of the fustis. They added an important finding about the constant presence of this smooth bony plate in the 42 subjects that were examined. In all cases, the fustis arose from the styloid prominence and coursed anteriorly as well as superiorly, pointing to the round window membrane itself (in more than 71.4%) or to its anterior edge (28.6%). The two types of fustis led to the scala tympani, which confirms that this structure is a robust and constant landmark during challenging cochlear implantation procedures. The mean angle between the fustis orientation and the central axis of the scala tympani was 155.4° (SD: 16.2) in Anschuetz et al. [28]. So far, and to the best of our knowledge, there are no studies describing fustis dimensions.

The second constant anatomical characteristic of the RW region relates to the 3D properties of the RW membrane. Indeed, it should not be looked at as a flat ovoid, round, or triangular membrane. There is a 3D feature of the RW membrane common to all two-dimensional shapes, which is its conical aspect. In studies by Okuno & Sando [13] or Roland et al. [18], the description of a posterosuperior horizontal part and anteroinferior vertical part aims to account for this conical shape. The surface area of the vertical portion is slightly larger compared to the horizontal one and is used as the portal for cochlear implant insertion. In contrast, the horizontal segment of the membrane is in close vicinity to the osseous spiral lamina, which increases the risk of trauma transmitted to the inner ear in case of manipulation at that level. Conversely, Goycoolea & Lunman [29] and Carpenter et al. [30] emphasized the complex 3D structure of the RW membrane, which combines a succession of concave and convex areas. Liang et al. [22] measured the 3D surface of the RW membrane in 14 temporal bones using a micro-fringe projection system and confirmed this complex pattern. The 3D characteristics of the RW membrane were considered when the first RW coupler was developed by Vibrant MED-EL (Innsbruck, Austria) to optimize the coupling between the FMT and a conical RW membrane.

### 4.3. Implications for Future RW Devices

RW focuses much interest from researchers and clinicians because it imposes itself as the optimal vehicle in the middle ear cleft for treatments which target the inner ear, whether they involve drugs or medical devices. Indeed, the RW membrane is constant (apart from exceptional cases of RW ossification), independent of ossicular status, and more accessible than the oval window, which is physiologically obstructed by the stapes presence. Composed of three different layers, it acts as a semipermeable membrane, which allows the passage of a wide range of drugs and vectors from corticosteroids (see Salt & Plontke for reviews [31]) to growth factors. Its mechanical contribution to hearing has been known since the mid-20th century and was popularized by Garcia-Ibanez under the concept of “sonoinversion” [32]. In cases of chronic otitis media with incus erosion, the tympanic membrane was medialized in contact with the RW membrane and protected by a piece of connective tissue. This procedure could improve air-conduction thresholds by up to 20 dB HL.

This systematic review of literature emphasized the anatomical variability of the RW area, and there is a significant proportion of cases where access to the RW membrane is spontaneously unfavorable due to the presence of a mucoperiosteal fold and/or a protruding tegmen. The lack of access has been suggested as a factor in the failure of intratympanic treatments [33,34,35]. High-resolution CT scans or transtympanic endoscopes have been used to anticipate such situations, and there are reports where access to the RW membrane was cleared to improve the potential efficacy of transtympanic treatments [36]. The procedure then requires the exposition of the RW by drilling a hypertrophic tegmen and removing the mucosal fold covering the membrane.

This strategy refers to the standardization of a variable anatomy to provide appropriate treatments in terms of size. The variability of the RW membrane measurements made from the scala tympani side in Liang et al. [22] is reduced compared to other studies, which suggests that this standardization is relevant to some extent. Plontke et al. [37] used an implant allowing a sustained release of dexamethasone to the RW membrane, divided into pieces of 0.8 mm to 1.5 mm each, as a function of RW membrane dimensions. Similarly, future developments of RW microperforators [38] or biodegradable reservoirs such as Hybrid Ear Cube will require this kind of procedure to improve access to the RW membrane. With regard to the dimensions of these devices, the 100 or 150 µm size of the microperforator tip is obviously compatible with the size range of the RW membrane. The two models of Hybrid Ear Cube (respectively, 0.6 mm and 0.8 mm maximum dimension) assessed in the proof-of-concept study fit within the RW niche [39]. In the case of RW vibroplasty, the dimensions of the FMT in the Vibrant Soundbridge active middle ear implant (Vibrant MED-EL, Innsbruck, Austria) seem more problematic. The diameter of the FMT (1.8 mm) exceeds a significant proportion of the RW membrane’s maximum dimensions. Based on studies included in this review where at least one maximum dimension of the RW membrane was reported at the individual level [19,20,22], the FMT placement in exclusive contact with the membrane would have been possible in only 12/54 (22%) of the total number of temporal bones. This means that the debate on the necessity of whether or not to drill the bony overhang of the RW in order to correctly place the FMT has a simple answer: it depends on the RW niche the surgeons have to deal with. Even if the bony overhangs are drilled, some patients with the FMT on an excessively small RW probably only benefit from the bone conduction transmitted to the bony edges [40]. This allows the correction of a conductive component involved in hearing loss, but should not have any impact on the sensorineural part. To fit the vast majority of RW membranes, the use of the RW coupler recently developed by MED-EL is mandatory, and in fact, an ideal transducer should measure 1 mm in diameter or less.

When facing the anatomical variability of the RW region, the alternative strategy is to adapt to it and develop individualized devices. Indeed, Matin et al. used Cone Beam Computed Tomographic reconstructions of the RW niche and membrane [15] to prepare 3D impressions of individualized drug-releasing implants. In this case, each patient would therefore receive a unique RW implant, made based on his/her own anatomical characteristics. Likewise, individualized RW couplers could be adapted to an optimized transducer in active middle ear implants.

### 4.4. Limitations

The main limitation of the study relates to the heterogeneity of the measurements, which were made across the 16 studies included, further reducing the number of studies with comparable data. Second, most studies were classified as “high risk of bias” according to the AQUA tool analysis because of the spatial resolution, the methods and/or techniques used to perform the measurements, or because only partial measurements were reported.

## 5. Conclusions

The variability in RW dimensions mainly relies on the relative development of the RW pillars and tegmen, which can be drilled to enhance RW membrane visualization along at least 1 mm of its minimum dimension. The fustis is a constant structure which corresponds to the floor of the RW niche and leads to the anterior part of the RW membrane. Future studies on RW membrane dimensions should consider measurements of the area, of the maximal and minimal diameters, before and after optimal drilling of the bony overhangs surrounding the RW membrane.

## Data Availability

No new data were created in this systematic review. Data sharing is not applicable to this article.

## Abbreviations

The following abbreviations are used in this manuscript:

CT	Computed Tomography
FMT	Floating Mass Transducer
RW	Round Window
